# CT findings in patients with Cabazitaxel induced pelvic pain and haematuria: a case series

**DOI:** 10.1186/s40644-017-0119-3

**Published:** 2017-06-24

**Authors:** Geethal N. Malalagama, Steve Chryssidis, Francis X Parnis

**Affiliations:** 1 Department of Medical Imaging, Flinders Medical Centre, Bedford Park, South Australia Australia; 2 Department of Oncology, Adelaide Cancer Centre, Kuralta Park, South Australia Australia

**Keywords:** Cabazitaxel, Haematuria, Pelvic, Pain, Dysuria

## Abstract

**Background:**

Haematuria and pelvic pain are recognized and documented adverse reactions related to Cabazitaxel use. To date there has not been any documentation of imaging findings in patients with this presentation.

**Cases:**

We report a case series of five patients who experienced these symptoms while on Cabazitaxel and were all found to have very similar urothelial changes on CT. The patients were noted to have ureteric and renal pelvic dilatation along with urothelial enhancement (in those who had post contrast imaging). All of these changes were noted to be reversible in those who had follow up imaging after cessation of Cabazitaxel and initiation of a short course of steroids.

**Conclusion:**

This case series helps demonstrate the pathological reversible urothelial inflammatory changes that may be occurring in patients experiencing haematuria and pelvic pain on Cabazitaxel therapy. These changes may relate to direct toxic effect of drug metabolites, a radiation recall type phenomenon or a combination of both.

## Background

Cabazitaxel is an antineoplastic agent currently indicated for the treatment of adult patients with hormone refractory metastatic prostate cancer, who have previously been treated with a Docetaxel containing regime. It works primarily through disruption of the microtubular network in cells which in turn inhibits mitotic and interphase cellular functions. Once administered intravenously Cabazitaxel is primarily metabolized in the liver (>95%) by mainly CYP3A isoenzyme (up to 90%). The majority of excretion is in faeces as numerous metabolites with less than 4% of the dose excreted by the kidneys [1].

The incidence of haematuria and pelvic pain with Cabazitaxel therapy is estimated to be 16.7% and 1.9% respectively [1].

We report a series of five cases of Cabazitaxel induced pelvic pain and microscopic haematuria with characteristic pattern of urothelial inflammatory changes on CT at the time of symptoms. All patients had a history of prior pelvic radiotherapy, uncomplicated treatment with Docetaxel and exclusion of urinary tract infections at the time of presentation.

## Case presentation

All patients in our series tolerated at least 5 cycles of Cabazitaxel prior to onset of symptoms, which included a varying description of pelvic pain with microscopic haematuria. Table 1 shows a clinical summary for each patient (cases 1–5).

**Table 1:** Summary of symptoms, relevant clinical history and management for each patient in our series

Case number	1	2	3	4	5
Symptoms	Lower pelvic and left flank pain	Lower abdominal and pelvic pain	Pelvic pain	Pelvic pain and dysuria	Pelvic pain
Radiotheraphy	64Gy in 32 fractions to whole pelvis in 2007	64Gy in 32 fractions to whole pelvis in 1996	70Gy in 35 fractions to whole pelvis in 2010	74Gy in 37 fractions to whole pelvis in 2006	20Gy in 5 fractions to right hemi-pelvis in 2015
Docetaxal theraphy	7 doses starting March 2015	5 doses starting October 2014	7 doses starting August 2013	6 doses starting July 2015	8 doses starting April 2016
Cabzitaxel theraphy	7 doses starting October 2015	5 doses starting May 2015	5 doses starting July 2014	7 doses starting March 2015	9 doses starting May 2016
Symptom management	Cessation of Cabazitaxel and short course of prednisolone	Cessation of cabazitaxel and short course of dexamethasone/prednisoloe	Cessation of cabazitaxel and short course of dexamethasone	Cessaiton of cabazitaxel without steroid theraphy	Cessaiton of cabazitaxel without steroid theraphy
Cabazitaxel re-initiation	Successful without recurrent symptoms	Not attempted	Not attempted	Not attempted	Successful without recurrent symptoms

**Fig. 1:**
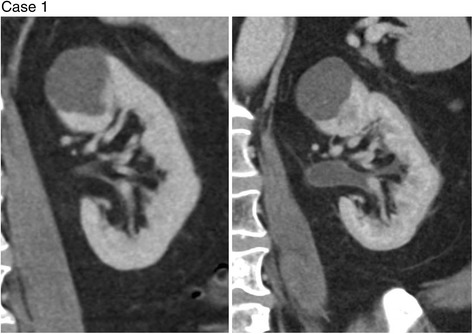
Coronal images of the left renal collecting system during Cabazitaxel treatment (*right*) shows significant dilatation and urothelial enhancement compared to pre Cabazitaxel treatment images (*left*)

Four of the patients in the series (cases 1–4) had whole pelvis radiotherapy and one patient (case 5) had radiotherapy only to the right hemi-pelvis (none had abdominal radiation). Corresponding to this, cases 1–4 (see Figs. 1 2 3 and 4) showed urothelial inflammatory changes bilaterally on CT, while case 5 (see Fig. 5) showed urothelial inflammation only on the right side.

**Fig. 2:**
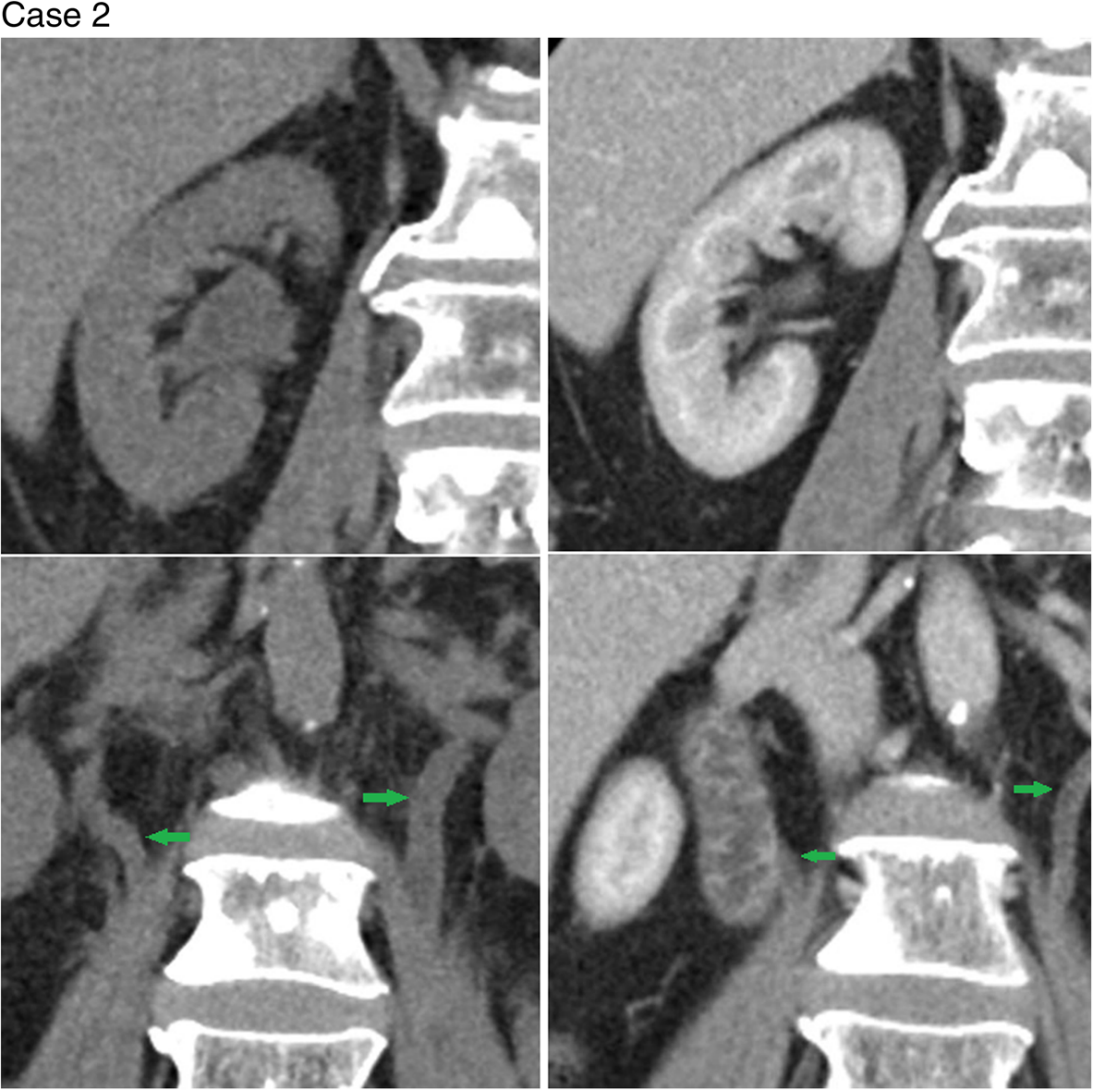
Coronal images of the right kidney (*top left*) and bilateral ureters (*bottom left - green arrows*) during Cabazitaxel therapy show dilatation compared to pre Cabazitaxel treatment (*top* and *bottom right*)

**Fig. 3:**
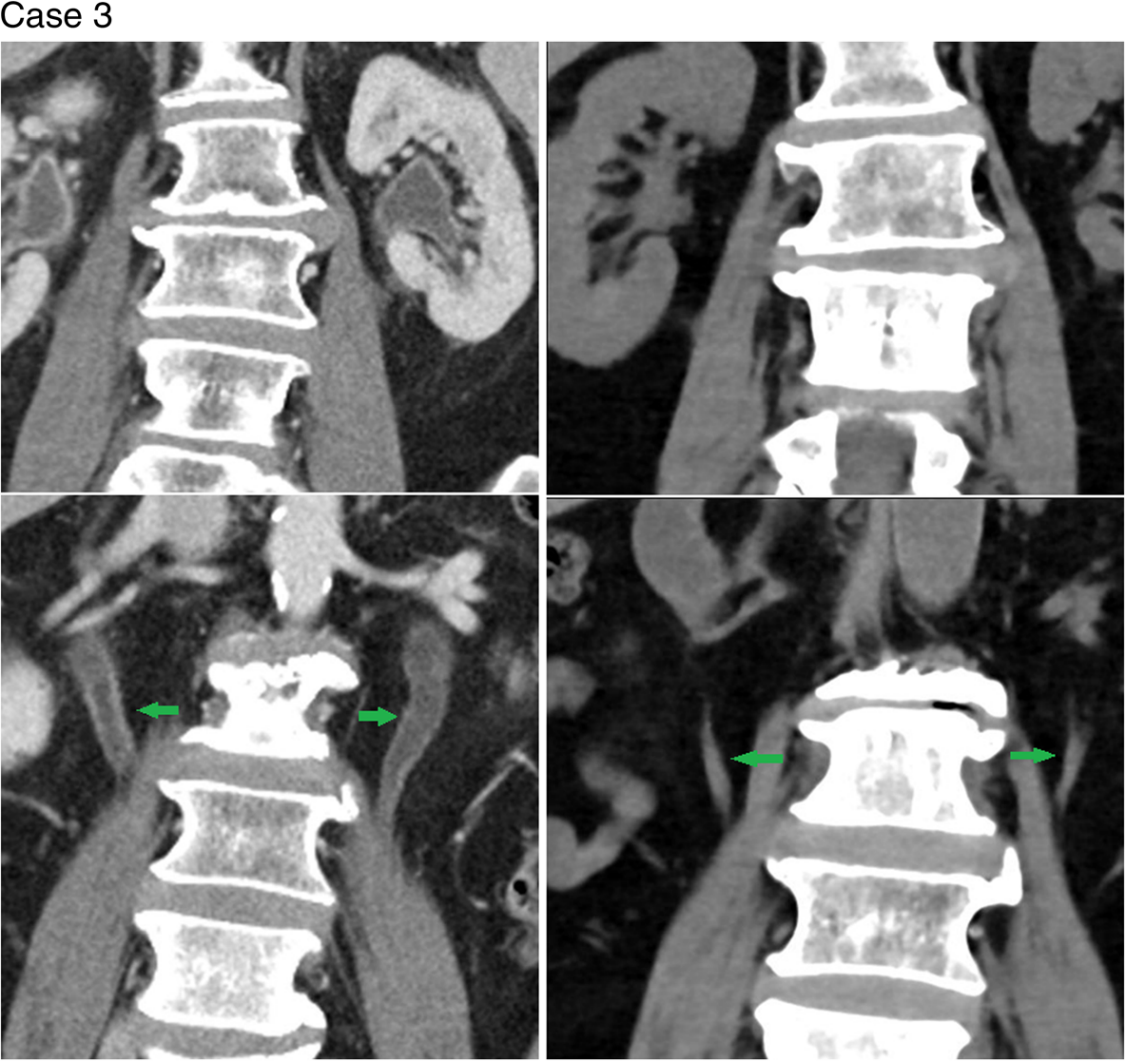
Coronal images of both kidneys (*top left*) and ureters (*bottom left - green arrows*) during Cabazitaxel therapy shows dilatation and urothelial enhancement. These changes have completely resolved 3 months after cessation of Cabazitaxel (*top* and *bottom right*)

**Fig. 4:**
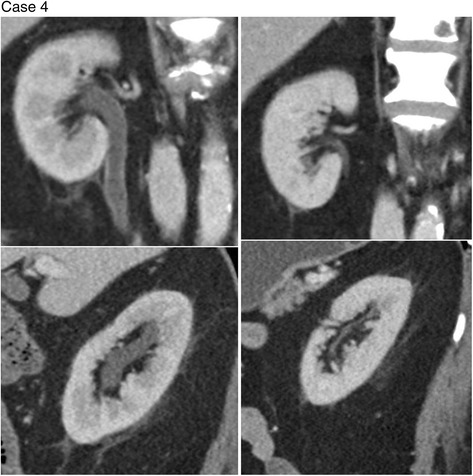
Coronal images of the right kidney (*top left*) and left kidney (*bottom left*) during Cabazitaxel therapy shows dilatation and urothelial enhancement. These changes had completely resolved at 5 months after Cabazitaxel cessation (*top* and *bottom right*)

**Fig. 5:**
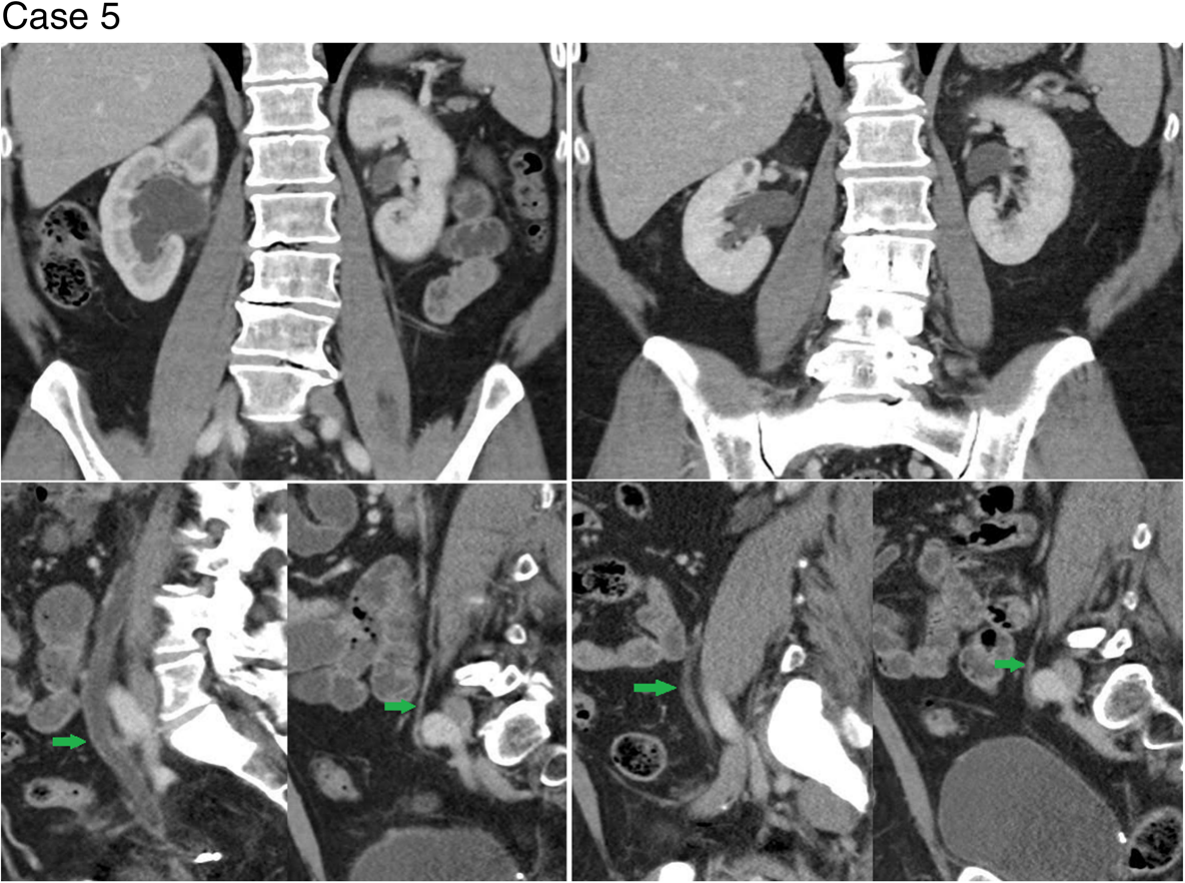
Coronal images of both kidneys (*top left*) and ureters (*bottom left - green arrows*) during Cabazitaxel treatment shows dilatation of the right ureter and renal pelvis with urothelial enhancement. The left ureter and renal pelvis appear unremarkable. Changes involving the right urothelium are most apparent when compared to pre Cabazitaxel treatment images of the kidneys (*top right*) and ureters (*bottom right - green arrows*)

Three out of the five patients in this group had improvement of symptoms with cessation of Cabazitaxel and initiation of a short course of steroid therapy, while the other two patients improved with Cabazitaxel cessation alone. Out of all five patients, two (one who had steroid therapy and one who did not) were able to be restarted on Cabazitaxel therapy without recurrence of significant symptoms.

Follow up imaging was conducted on two of the patients (cases 3 and 4) after resolution of pelvic pain and haematuria. These scans revealed significant improvement in the urothelial inflammatory changes supporting the reversible nature of these effects both clinically and radiologically.

## Discussion

Through this case series we are providing imaging based evidence of reversible urothelial inflammatory changes occurring in patients experiencing pelvic pain and haematuria while on Cabazitaxel therapy.

While reversible side effects such as pelvic pain and haematuria have been documented related to the use of Cabazitaxel [1], to our knowledge this is the first time that such extensive reversible urothelial inflammatory changes have been noted on imaging.

We hypothesise these changes to be due to either direct toxic effect of Cabazitaxel (or its metabolites) on the urothelium or the provocation of radiation recall syndrome or a combination of both.

The reversibility of inflammatory changes following Cabazitaxel cessation is compatible with both drug toxicity and radiation recall syndrome [2]. Very limited renal excretion of Cabazitaxel/metabolites [1], attenuated recurrence of symptoms following re-initiation [2, 3] and the presence of trial data showing positive correlation between pelvic radiation and haematuria [4, 5] in patients treated with Cabazitaxel, however, all favour this to be related to radiation recall.

We feel that the unilateral nature of urothelial inflammatory changes in case 5 (corresponding to the side of hemi-pelvic radiation) also further supports the possibility of a radiation recall syndrome rather than drug toxicity (where one would expect inflammation to be bilateral in all cases).

Although the timing of symptom onset to beyond 5 cycles of treatment and involvement of the entire urothelium rather than just the radiated pelvic regions is atypical for radiation recall syndrome, it is still possible for such a presentation to occur [2, 3, 6]. Certainly the extension of inflammatory change beyond the original radiation field is an accepted entity [6, 7].

There is at least one case series we are aware of that has histologically shown urothelial inflammation from Cabazitaxel induced radiation recall syndrome [8]. Whilst our case series exhibits some atypical features we feel the most likely cause for these inflammatory change is a radiation recall syndrome.

It must be noted that if this is indeed a radiation recall syndrome while successful re-initiation is possible (as in our case series), there is also the possibility of severe symptom recurrence at re-initiation [6] and caution must be taken.

We hope our observations will help to improve assessment and management of patients experiencing pelvic pain and haematuria while on Cabazitaxel treatment.

## Abbreviation

CT: computed tomography
